# A Note on Cumulant Technique in Random Matrix Theory

**DOI:** 10.3390/e25050725

**Published:** 2023-04-27

**Authors:** Alexander Soshnikov, Chutong Wu

**Affiliations:** Department of Mathematics, University of California at Davis, Davis, CA 95616, USA; ctwu@ucdavis.edu

**Keywords:** random matrices, cumulants, Central Limit Theorem

## Abstract

We discuss the cumulant approach to spectral properties of large random matrices. In particular, we study in detail the joint cumulants of high traces of large unitary random matrices and prove Gaussian fluctuation for pair-counting statistics with non-smooth test functions.

## 1. Introduction

Random Matrix Theory has its origins in the works of statisticians in the 1920s and nuclear physicists in the 1950s. In the pioneering papers [1,2,3], Eugene Wigner introduced an ensemble of random matrices that now have his name and computed the limiting spectral distribution. The main ingredient of the proof was the method of moments that allowed Wigner to study asymptotics of the traces of powers of a random symmetric (Hermitian) matrix with independent identically distributed (i.i.d.) components. Since then, the method of moments has been successfully used to study spectral properties of large random matrices on many occasions. It works particularly well when a random matrix has many independent components. We refer the reader to [4,5,6,7,8,9,10,11,12,13] and references therein.

The purpose of this paper is to discuss several applications of the cumulant technique in Random Matrix Theory. The cumulant approach is especially useful if point correlation functions are given by the determinantal or Pfaffian formulas.

The paper is organized as follows. In the Methods section, we give a brief overview of the known results. Several novel results related to the joint cumulants of traces of high powers and pair counting statistics of eigenvalues of a large random unitary matrix are formulated and proved in the Results section. The Discussion section is devoted to brief comments on the future lines of research.

Throughout the paper, the notation $a_{N}=O\left(b_{N}\right)$ means that the ratio $a_{N}/b_{N}$ is bounded from above in absolute value. The notation $a_{N}=o\left(b_{N}\right)$ means that $a_{n}/b_{N}\to 0$ as N→∞. The notation $a_{N}=\Omega \left(b_{N}\right)$ means that the ratio $a_{N}/b_{N}$ is bounded from above in absolute value by a power of logN. Finally, we sometimes use the notation a∨b for the maximum of *a* and b.

## 2. Methods

### 2.1. Determinantal Point Process and Cluster Functions

The ideas of the cumulant approach in Random Matrix Theory go back at least to the 1995 paper [14] by Costin and Lebowitz, which studied a so-called sine random point process, namely, a determinantal random point process with the correlation kernel

(1) $$K\left(x,y\right)=\frac{\sin(\pi \,(x-y))}{\pi \,(x-y)}.$$

In other words, the *k*-point correlation functions of the random point process are given by

(2) $$\rho_{k}\left(x_{1},\ldots ,x_{k}\right)=\det{\left[K\left(x_{i},x_{j}\right)\right]}_{1\leq i,j\leq k},\;\;\;\;k\geq 1.$$

We refer the reader to [15,16] for an introduction to determinantal random point processes. The sine random point process appears as a scaling limit of many ensembles of random matrices, including the Circular Unitary Ensemble defined below in (11).

The main result of [14] states that #[0,L], the number of particles in an interval [0,L], has Gaussian fluctuation in the limit L→∞ with logarithmically growing variance. Namely,

(3) $$\frac{\#[0,L]-L}{\sqrt{\log L}}\overset{\;D\;}{\to }N\left(0,1/\pi^{2}\right).$$

To study the limiting distribution of the counting random variable #[0,L], Costin and Lebowitz suggested using the so-called cluster (Ursell) *k*-point functions. For k=1,2,3, the cluster functions are given by

$$\begin{array}{cc} \; & r_{1}\left(x_{1}\right)=\rho_{1}\left(x_{1}\right),\\ \; & r_{2}\left(x_{1},x_{2}\right)=\rho_{2}\left(x_{1},x_{2}\right)-\rho_{1}\left(x_{1}\right)\,\rho_{1}\left(x_{2}\right),\\ \; & r_{3}\left(x_{1},x_{2},x_{3}\right)=\rho_{3}\left(x_{1},x_{2},x_{3}\right)-\rho_{2}\left(x_{1},x_{2}\right)\,\rho_{1}\left(x_{3}\right)-\rho_{2}\left(x_{1},x_{3}\right)\,\rho_{1}\left(x_{2}\right)-\\ \; & -\rho_{2}\left(x_{2},x_{3}\right)\,\rho_{1}\left(x_{1}\right)+2\,\rho_{1}\left(x_{1}\right)\,\rho_{1}\left(x_{2}\right)\,\rho_{1}\left(x_{3}\right). \end{array}$$

For arbitrary k≥1, one has

(4) $$r_{k}\left(x_{1},\ldots ,x_{k}\right)=\sum_{\pi }{\left(-1\right)}^{|\pi |-1}\,\left(|\pi |-1\right)!\,\prod_{j=1}^{\left|\pi \right|}\rho_{|B_{j}|}\left(x_{i}:i\in B_{j}\right),$$

where the sum at the r.h.s. of (4) is over all partitions π of the set {1,2,…,k} into blocks $B_{1},\ldots ,B_{m},\;\;\left|\pi \right|=m$ stands for the number of blocks in a partition π, and $|B_{i}|$ denotes the cardinality of a block $B_{i}.$

For determinantal random point processes, (2) and (4) imply

$$\begin{array}{ccc} r_{k}\left(x_{1},\ldots ,x_{k}\right) & = & \frac{{\left(-1\right)}^{k-1}}{k}\,\sum_{\sigma \in S_{k}}K\left(x_{\sigma (1)},x_{\sigma (2)}\right)\,K\left(x_{\sigma (2)},x_{\sigma (3)}\right)\,\ldots \,K\left(x_{\sigma (k)},x_{\sigma (1)}\right)\\ \; & = & {\left(-1\right)}^{k-1}\,\left(K\left(x_{1},x_{2}\right)\,K\left(x_{2},x_{3}\right)\,\ldots \,K\left(x_{k},x_{1}\right)+\ldots \right),\;\;\;\;\;\;\;\;\;\;\;\;\;\;\;\;\;\;\;\; \end{array}$$

where the sum in the first line is over all permutations in the symmetric group $S_{k}$, and the sum in the second line is over all (k−1)! cyclic permutations in $S_{k}$, with the first of such cyclic permutations being 1→2→3→…→k→1.

Cluster point functions are closely related to the cumulants of #[0,L]. Denote by $I_{k}\left(L\right)$ the integral of the *k*-point cluster function over the *k*-dimensional cube ${\left[0,L\right]}^{k}:$

(5) $$I_{k}\left(L\right)=\int_{{\left[0,L\right]}^{k}}r_{k}\left(x_{1},\ldots ,x_{k}\right)\,dx_{1}\ldots dx_{k},\;\;k\geq 1.$$

Furthermore, denote by $\kappa_{j}\left(L\right),\;j=1,2,3,\ldots ,$ the cumulants of the counting random variable #[0,L]. Recall that the moment and cumulant generating functions are related by

(6) $$\sum_{j=1}^{\infty }\frac{\kappa_{j}}{j!}\,z^{j}=\log(\sum_{j=0}^{\infty }\frac{m_{j}}{j!}\,z^{j}).$$

It follows from (4) and (6) that the *j*-th cumulant of #[0,L] can be written as a linear combination of the integrals $I_{k}\left(L\right),\;1\leq k\leq j.$ Namely, the following relation holds for the generating functions:

(7) $$\sum_{j=1}^{\infty }\frac{\kappa_{j}\left(L\right)}{j!}\,z^{j}=\sum_{j=1}^{\infty }\frac{I_{j}\left(L\right)}{j!}\,{\left(e^{z}-1\right)}^{j}.$$

To prove the Central Limit Theorem for the normalized random variable $\frac{\#[0,L]-E\#[0,L]}{\sqrt{\mathrm{Var}\#[0,L]}}$, it is sufficient to show that

(8) $$\mathrm{Var}\#\left[0,L\right]\to \infty \;\;as\;\;L\to \infty \;\;and\;\;\kappa_{j}\left(L\right)=o\left({\left(\mathrm{Var}\#\left[0,L\right]\right)}^{j/2}\right)\;\;for\;\;j>2$$

since (8) would imply that all cumulants starting with the third one of the normalized counting random variable $\frac{\#[0,L]-E\#[0,L]}{\sqrt{\mathrm{Var}\#[0,L]}}$ go to zero as L→∞.

It is not hard to see that if a random point process is such that Var#[0,L] grows linearly in *L* and the cluster function integrals satisfy

(9) $$I_{j}\left(L\right)=o\left(L^{j/2}\right),\;\;\;j>2,$$

a routine application of (7) and (8) finishes the proof of the Central Limit Theorem for the counting function. However, the sine random point process exhibits a more delicate behavior—the variance of the number of particles grows only logarithmically, namely:

(10) $$\mathrm{Var}\#\left[0,L\right]=\frac{1}{\pi^{2}}\,\log L+O\left(1\right).$$

Thus, a more subtle analysis of the asymptotics of $I_{j}\left(L\right)$ is required for j>2. The proof ([14]) of the Central Limit Theorem (3) follows from the bounds

$$\begin{array}{c} \int_{0}^{L}\,\int_{0}^{L}\,\frac{\sin(\pi \left(x_{1}-x_{2}\right))}{\pi \,(x_{1}-x_{2})}\,\frac{\sin(\pi \,\left(x_{2}-x_{1}\right))}{\pi \,(x_{2}-x_{1})}\;dx_{1}\,dx_{2}=L-\frac{1}{\pi^{2}}\,\log L+O\left(1\right),\\ \int_{{\left[0,L\right]}^{k}}\frac{\sin(\pi \,\left(x_{1}-x_{2}\right))}{\pi \,(x_{1}-x_{2})}\,\frac{\sin(\pi \,\left(x_{2}-x_{3}\right))}{\pi \,(x_{2}-x_{3})}\,\ldots \,\frac{\sin(\pi \,\left(x_{k}-x_{1}\right))}{\pi \,(x_{k}-x_{1})}\;dx_{1}\cdots dx_{k}=L+O\left(\log L\right). \end{array}$$

Remarkably, the result can be generalized to the case of any determinantal random point field on a locally compact Hausdorff phase space *E*, equipped with a σ-finite measure μ on the Borel σ-algebra provided the correlation kernel is Hermitian and locally trace class and the variance goes to infinity. Namely, the following holds:

**Theorem 1**  ([17,18])**.** *Let $\left(X,F,P_{n}\right)$ be a family of determinantal random point fields with hermitian locally trace class correlation kernels $K_{n}$ and $I_{n}$ be a family of Borel subsets of E with compact closure. Then, if ${Var}_{n}\#\left(I_{n}\right)\to \infty$ as n→∞, the normalized counting random variable $\frac{\#\left(I_{n}\right)-E\#\left(I_{n}\right)}{\sqrt{Var\#(I_{n})}}$ converges in distribution to a standard normal N(0,1).*

**Remark 1.**  *The multivariate version of the theorem also holds (see [17,18]). It is worth noting that the univariate result allows a very nice probabilistic interpretation ([19]). Namely, one can show that the counting random variable #(I) is equal in distribution to a sum of independent non-identically distributed Bernoulli random variables with the probabilities of success given by the eigenvalues of the integral operator $K_{I}f\left(x\right)=\int_{I}K\left(x,y\right)\,f\left(y\right)\,\mu \left(dy\right)\;$ on $L^{2}\left(I,\mu \right).\;$ However, we are not aware of a simple probabilistic interpretation of the multivariate CLT result.*

Analogous results hold for linear statistics $L_{f}=\sum_{i}f\left(x_{i}\right),$ where *f* is a bounded measurable test function, e.g., with a compact support, and the summation is over all points of a random point field.

**Theorem 2**  ([17,18])**.** *Let $\left(X,F,P_{n}\right)$ be a family of determinantal random point fields with hermitian locally trace class correlation kernels $K_{n}$ and $f_{n}$ be a bounded, measurable, compactly supported function on the phase space E such that*

$$VarL_{f_{n}}\to \infty ,\;\;\sup\left|f_{n}\left(x\right)\right|=o\left({\left(VarL_{f_{n}}\right)}^{\varepsilon }\right),\;\;EL_{{\left|f\right|}_{n}}=O\left({\left(VarL_{f_{n}}\right)}^{\delta }\right),$$

*for any ε>0 and some δ>0 as n→∞. Then, the normalized linear statistic $\frac{L_{f_{n}}-EL_{f_{n}}}{\sqrt{VarL_{f_{n}}}}$ converges in distribution to N(0,1) as n→∞.*

For additional results in this direction, we refer the reader to [20,21,22]. Moreover, the connection between integrals of cluster point functions and cumulants can be exploited to study significantly more challenging problems regarding empirical spectral distribution of spacings and extreme spacings in determinantal random point processes (see, e.g., [23,24]).

### 2.2. Linear Statistics in Classical Compact Groups

For many ensembles of large random matrices, the variance of a linear statistic either stays finite or grows slower than any power of the dimension, provided the test function is sufficiently smooth. Therefore, Theorem 2 is not typically applicable even if the point correlation functions are determinantal. It is instructive to consider classical compact groups. In this paper, we focus our attention on random unitary matrices. However, many results can be extended to the orthogonal and symplectic matrices without much difficulty.

Let *V* be a unitary matrix chosen at random with respect to the Haar measure on the unitary group U(N). We are interested in studying statistical properties of the eigenvalues of *V*, which we denote by $e^{i\,\theta_{j}},\;1\leq j\leq N,\;-\pi \leq \theta_{1},\ldots ,\theta_{N}<\pi .\;$ The joint probability density of the eigenvalues is given by the formula ([25]):

(11) $$\begin{array}{c} p_{N}\left(\theta_{1},\ldots ,\theta_{N}\right)=\frac{1}{{\left(2\,\pi \right)}^{N}\,N!}\,\prod_{1\leq j<k\leq N}{\left|e^{i\theta_{j}}-e^{i\theta_{k}}\right|}^{2}.\;\;\; \end{array}$$

The joint distribution of the eigenvalues is known as the Weyl measure, and the ensemble is known in Random Matrices as the Circular Unitary Ensemble ([26,27,28]). Remarkably, if a test function *f* on the unit circle is sufficiently smooth, the variance of the linear statistics $\sum_{j=1}^{N}f\left(\theta_{j}\right)$ stays finite as N→∞. Moreover,

**Theorem 3**  ([29,30])**.** *Let f be a real-valued function on the unit circle satisfying*

(12) $$\sum_{-\infty }^{\infty }|\hat{f_{k}}{|}^{2}\,\left|k\right|<\infty ,$$

*where*

(13) $$\hat{f_{k}}=\frac{1}{2\,\pi }\,\int_{-\pi }^{\pi }f\left(x\right)\,e^{-i\,k\,x}\,dx,\;\;k\in Z$$

*are the Fourier coefficients of f. Then,*

(14) $$\sum_{j=1}^{N}f\left(\theta_{j}\right)-\hat{f_{0}}\,N\overset{\;D\;}{\to }N(0,\sum_{-\infty }^{\infty }|\hat{f_{k}}{|}^{2}\,|k|).$$

**Remark 2.**  *One can show that the result of Theorem 3 follows from the Strong Szego Theorem for Toeplitz determinants ([30]).*

**Remark 3.**  *It follows from Theorem 3 that the real and imaginary parts of the traces of the powers of a random unitary matrix, $\mathrm{Tr}V^{k}=\sum_{j=1}^{N}e^{i\,k\,\theta_{j}},\;k\geq 1,$ converge to independent normal random variables with mean zero and variance k/2 as N→∞. In ([31]), Johansson proved that the rate of convergence to normal law is $O(N^{-\delta \,N})$ for some δ>0. Recently, the results have been further improved in [32,33].*

Many joint moments of the traces of powers of a random unitary matrix *V* coincide with the corresponding joint moments of the limiting normal random variables. Namely, let $Z_{j},\;j\geq 1,$ be a sequence of independent standard complex normals. Then, for any k≥1 and non-negative integers $a_{j},b_{j},1\leq j\leq k$ satisfying

(15) $$N\geq \left(\sum_{j=1}^{k}j\,a_{j}\right)\vee \left(\sum_{j=1}^{k}j\,b_{j}\right),$$

one has ([29]):

(16) $$E\left[\prod_{j=1}^{k}\mathrm{Tr}{\left(V^{j}\right)}^{a_{j}}\bar{\mathrm{Tr}{\left(V^{j}\right)}^{b_{j}}}\right]=\delta_{ab}\prod_{j=1}^{k}j^{a_{j}}a_{j}!=E\left[\prod_{j=1}^{k}{\left(\sqrt{j}Z_{j}\right)}^{a_{j}}\bar{{\left(\sqrt{j}Z_{j}\right)}^{b_{j}}}\right],$$

where the delta symbol $\delta_{ab}$ is one if $a=(a_{1},\ldots ,a_{k})$ coincides with $b=(b_{1},\ldots ,b_{k})$ and is zero otherwise. For a beautiful survey of this and related results for random matrices from classical compact groups, we refer the reader to [34].

To better understand (16) and related phenomena, one can study the joint cumulants of the traces of powers of a random matrix. Let $\left\{X_{\alpha \in A}\right\}$ be a family of random variables. The joint cumulants are defined as (see, e.g., [35]):

(17) $$\begin{array}{c} \kappa_{i_{1},\ldots ,i_{n}}:=\kappa \left(X_{i_{1}},\ldots ,X_{i_{n}}\right)=\sum_{\pi }\left(|\pi |-1\right)!{\left(-1\right)}^{|\pi |-1}\prod_{B\in \pi }E\left(\prod_{i\in B}X_{i}\right), \end{array}$$

where the sum is over all partitions π of the set $\{i_{1},\ldots ,i_{n}\},$ *B* goes over the list of all blocks of the partition π, and |π| denotes the number of blocks in the partition.

We recall that the joint moments are expressed in terms of the cumulants as

(18) $$\begin{array}{c} E\left(X_{1},\ldots ,X_{n}\right):=E\prod_{1\leq i\leq n}X_{i}=\sum_{\pi }\prod_{B\in \pi }\kappa \left(X_{i}:i\in B\right). \end{array}$$

We will use the notation $T_{N,k}:=\mathrm{Tr}\left(V^{k}\right)$ for the traces of powers of *V* and denote by $\kappa_{p}^{\left(N\right)}\left(k_{1},\ldots ,k_{p}\right)$ the joint cumulants of $T_{N,k_{1}},\ldots ,T_{N,k_{p}},$ i.e.,

(19) $$\kappa_{p}^{\left(N\right)}\left(k_{1},\ldots ,k_{p}\right):=\kappa \left(T_{N,k_{1}},\ldots ,T_{N,k_{p}}\right).$$

For a determinantal random point process with a correlation kernel K(x,y), the cumulants of a linear statistic $L_{f}=\sum_{i}f\left(x_{i}\right)$ can be computed as ([18]):

(20) $$\begin{array}{c} \kappa_{p}\left(L_{f}\right)=\sum_{m=1}^{p}\,\frac{{\left(-1\right)}^{m}}{m}\,\sum_{\begin{array}{c} (p_{1},\ldots ,p_{m}):\\ p_{1}+\ldots +p_{m}=p,\;p_{1},\ldots ,p_{m}\geq 1 \end{array}}\frac{p!}{p_{1}!\cdots p_{m}!}\times \end{array}$$

(21) $$\begin{array}{c} \int f^{p_{1}}\left(x_{1}\right)\,K\left(x_{1},x_{2}\right)\,f^{p_{2}}\left(x_{2}\right)\,K\left(x_{2},x_{3}\right)\cdots f^{p_{m}}\left(x_{m}\right)\,K\left(x_{m},x_{1}\right)\,dx_{1}\cdots dx_{m}. \end{array}$$

For the CUE, the point correlation functions are given by the determinantal Formula (2) with the correlation kernel

$$K_{N}\left(x,y\right)=\frac{1}{2\,\pi }\,\sum_{j=0}^{N-1}e^{i\,j\,(x-y)}.$$

This allows one to obtain the following formula in the CUE case, provided the test function *f* is sufficiently smooth ([36], see also [37,38,39]):

$$\begin{array}{c} \kappa_{p}\left(L_{f}\right)=\sum_{\begin{array}{c} k_{1},\ldots ,k_{p}\in Z\\ k_{1}+\ldots +k_{p}=0 \end{array}}\;\prod_{j=1}^{p}\;{\hat{f}}_{k_{j}}\;\sum_{m=1}^{p}\,\frac{{\left(-1\right)}^{m-1}}{m}\,\sum_{\begin{array}{c} (p_{1},\ldots ,p_{m}):\\ p_{1}+\ldots +p_{m}=p,\;p_{1},\ldots ,p_{m}\geq 1 \end{array}}\frac{p!}{p_{1}!\cdots p_{m}!}\times \\ \max\left(0,N-\max(0,\sum_{i=1}^{p_{1}}k_{i},\ldots ,\sum_{i=1}^{p_{1}+\ldots +p_{m-1}}k_{i})-\max(0,-\sum_{i=1}^{p_{1}}k_{i},\ldots ,-\sum_{i=1}^{p_{1}+\ldots +p_{m-1}}k_{i})\right), \end{array}$$

that can be rewritten as

(22) $$\begin{array}{c} \kappa_{p}\left(L_{f}\right)=\sum_{\begin{array}{c} k_{1},\ldots ,k_{p}\in Z\\ k_{1}+\ldots +k_{p}=0 \end{array}}\;\prod_{j=1}^{p}\;{\hat{f}}_{k_{j}}\;\sum_{m=1}^{p}\,\frac{{\left(-1\right)}^{m}}{m}\;\sum_{\begin{array}{c} (p_{1},\ldots ,p_{m}):\\ p_{1}+\ldots +p_{m}=p,\;p_{1},\ldots ,p_{m}\geq 1 \end{array}}\frac{1}{p_{1}!\cdots p_{m}!}\times \\ \sum_{\sigma \in S_{p}}\;J_{N}\left(p_{1},\ldots ,p_{m},k_{\sigma (1)},\ldots ,k_{\sigma (p)}\right), \end{array}$$

where $J_{N}$ is a piece-wise linear function defined by the following formula

(23) $$\begin{array}{cc} \; & J_{N}\left(p_{1},\ldots ,p_{m};k_{1},\ldots ,k_{p}\right):=\\ \; & \min\left(N,\;\max(0,\sum_{i=1}^{p_{1}}k_{i},\sum_{i=1}^{p_{1}+p_{2}}k_{i},\ldots ,\sum_{i=1}^{p_{1}+\ldots +p_{m-1}}k_{i})+\max(0,-\sum_{i=1}^{p_{1}}k_{i},\ldots ,-\sum_{i=1}^{p_{1}+\ldots +p_{m-1}}k_{i})\right) \end{array}$$

for positive integers $p_{1},\ldots ,p_{m}\geq 1,\;\;p_{1}+\ldots +p_{m}=p,$ and integers $k_{1},\ldots ,k_{p},$ satisfying $\sum_{i=1}^{p}k_{i}=0.\;$ Since the joint cumulants are symmetric and multilinear, it follows from (22) and (23) that $\kappa_{p}^{\left(N\right)}\left(k_{1},\ldots ,k_{p}\right)$ can be computed as

**Lemma 1**  ([40])**.**

*(i) If p>1 and either $k_{1}+\ldots +k_{p}\neq 0$ or $\;\prod_{i}^{p}k_{i}=0,\;$ or both, then*

(24) $$\begin{array}{c} \kappa_{p}^{\left(N\right)}\left(k_{1},\ldots ,k_{p}\right)=0. \end{array}$$

*(ii) If $p>1,\;\;\prod_{i}^{p}k_{i}\neq 0,$ and $\sum_{i=1}^{p}k_{i}=0,$ then*

(25) $$\begin{array}{c} \kappa_{p}^{\left(N\right)}\left(k_{1},\ldots ,k_{p}\right)=\sum_{m=1}^{p}\frac{{\left(-1\right)}^{m}}{m}\,\sum_{\begin{array}{c} (p_{1},\ldots ,p_{m}):\\ p_{1}+\ldots +p_{m}=p,\;p_{1},\ldots ,p_{m}\geq 1 \end{array}}\frac{1}{p_{1}!\cdots p_{m}!}\times \\ \sum_{\sigma \in S_{p}}\,J_{N}\left(p_{1},\ldots ,p_{m};k_{\sigma (1)},\ldots ,k_{\sigma (p)}\right). \end{array}$$

**Remark 4.**  *Alternatively, one can rewrite the last formula as*

(26) $$\begin{array}{c} \kappa_{p}^{\left(N\right)}\left(k_{1},\ldots ,k_{p}\right)=\sum_{m=1}^{p}\frac{{\left(-1\right)}^{m}}{m}\,\sum_{\begin{array}{c} (C_{1},\ldots ,C_{m}):\\ C_{1}\cup \ldots \cup C_{m}=\left\{1,\ldots ,p\right\} \end{array}}\;\;\;\;\;\;\;\;\;\;\;\;\;\;\;\;\;\;\;\;\;\;\;\;\;\;\;\;\;\;\;\;\;\;\;\;\;\;\;\;\;\;\;\;\;\;\;\\ \min\left(N,\;\max(0,\sum_{i\in C_{1}}k_{i},\ldots ,\sum_{i\in C_{1}\cup \ldots \cup C_{m-1}}k_{i})+\max(0,-\sum_{i\in C_{1}}k_{i},\ldots ,-\sum_{i\in C_{1}\cup \ldots \cup C_{m-1}}k_{i})\right), \end{array}$$

*where the summation is over all ordered collections of non-empty disjoint subsets* $C_{1},\ldots ,C_{m},\;$m≥1, *of the set {1,…,p} such that $\cup_{j}C_{j}=\left\{1,\ldots ,p\right\}.$*

It should be emphasized that the exact combinatorial structure manifested in Lemma 1 is specific to the classical compact groups and the sine determinantal point process.

To finish the proof of (14) and (16), using the cumulants, one observes that for

(27) $$\sum_{i=1}^{p}\left|k_{i}\right|\leq 2\,N\;and\;\sum_{i=1}^{p}k_{i}=0$$

the expression for $J_{N}\left(p_{1},\ldots ,p_{m};k_{1},\ldots ,k_{p}\right)$ simplifies, namely:

(28) $$\begin{array}{cc} \; & J_{N}\left(p_{1},\ldots ,p_{m};k_{1},\ldots ,k_{p}\right)=\\ \; & \max\left(0,\sum_{i=1}^{p_{1}}k_{i},\sum_{i=1}^{p_{1}+p_{2}}k_{i},\ldots ,\sum_{i=1}^{p_{1}+\ldots +p_{m-1}}k_{i}\right)+\max\left(0,\sum_{i=1}^{p_{1}}\left(-k_{i}\right),\ldots ,\sum_{i=1}^{p_{1}+\ldots +p_{m-1}}\left(-k_{i}\right)\right). \end{array}$$

One then uses a combinatorial lemma from [36] to show that all joint cumulants $\kappa_{p}^{\left(N\right)}\left(k_{1},\ldots ,k_{p}\right)$ of order three and higher (p>2) are identically zero, provided $\sum_{i=1}^{p}\left|k_{i}\right|\leq 2\,N$:

**Lemma 2**  ([36])**.** *Let $k_{1},\ldots ,k_{p},\;\;p\geq 2$ be arbitrary real numbers such that $\sum_{i}k_{i}=0.$ Then, the sum*

$$\sum_{\sigma \in S_{p}}\;\sum_{m=1}^{p}\,\frac{{\left(-1\right)}^{m}}{m}\,\sum_{\begin{array}{c} (p_{1},\ldots ,p_{m}):\\ p_{1}+\ldots +p_{m}=p,\;p_{1},\ldots ,p_{m}\geq 1 \end{array}}\frac{1}{p_{1}!\cdots p_{m}!}\,\max(0,\sum_{i=1}^{p_{1}}k_{\sigma (i)},\ldots ,\sum_{i=1}^{p_{1}+\ldots +p_{m-1}}k_{\sigma (i)})$$

*equals $|k_{1}\left|=\right|k_{2}|$ if p=2 and zero if p>2.*

**Remark 5.**  *The lemma is related to the combinatorial proof of the Strong Szego Theorem. We refer the reader to [41,42,43,44] for recent applications of the cumulant method for determinantal processes to study linear statistics of Hermitian unitary invariant random matrices and free fermions processes.*

### 2.3. Multivariate Linear Statistics and Number Theory Connections

This subsection is devoted to the discussion of multivariate linear statistics of the form

(29) $$\begin{array}{c} S_{N}\left(f\right)=\sum_{1\leq j_{1},j_{2},\ldots ,j_{n+1}\leq N}f\left(L_{N}\,\left(\theta_{j_{2}}-\theta_{j_{1}}\right),\ldots ,L_{N}\,\left(\theta_{j_{n+1}}-\theta_{j_{1}}\right)\right), \end{array}$$

where $\theta =\left(\theta_{1},\ldots ,\theta_{N}\right)\in T^{N}$ comes from the CUE (or, in general, the Circular Beta Ensemble with arbitrary β>0), the scaling factor $L_{N}$ satisfies $1\leq L_{N}\leq N,$ and *f* is a sufficiently smooth test function defined on the unit circle (when $L_{N}=1$) or the real line (when $L_{N}\to \infty$).

The study of such multivariate linear statistics in [40,45,46,47] was motivated by connections between Random Matrices and Number Theory (see, e.g., [48,49,50,51] and references therein). The local case ($L_{N}=N$) is of a particular interest for the CUE since the multivariate linear statistic

(30) $$\begin{array}{c} S_{N}\left(f\right)=\sum_{1\leq j_{1},j_{2},\ldots ,j_{n+1}\leq N}f\left(N\,{\left(\theta_{j_{2}}-\theta_{j_{1}}\right)}_{c},\ldots ,N\,{\left(\theta_{j_{n+1}}-\theta_{j_{1}}\right)}_{c}\right), \end{array}$$

where $f\in C_{c}^{\infty }\left(R\right)$ and ${\left(\theta -\phi \right)}_{c}$ is the phase difference on the unit circle (i.e., the difference modulo 2⁢π) and corresponds to multivariate linear statistics of the (rescaled) zeros of the Riemann zeta function studied by Montgomery [52,53], Hejhal [54], and Rudnick and Sarnak [55] (see, e.g., [40]). We also point out a recent related preprint [56], where multivariate linear statistics have been studied for the determinantal random process with the projection correlation kernel on the unit sphere $S^{d},\;d>1$.

To study the limiting distribution of (30), we write using the Fourier transform

(31) $$S_{N}\left(f\right)=\frac{1}{{\left(2\pi \right)}^{n/2}\,N^{n}}\,\sum_{k\in Z^{n}}\hat{f}(\frac{k_{1}}{N},\ldots ,\frac{k_{n}}{N})\,\prod_{j=1}^{n+1}T_{N,k_{j}}$$

where $k_{n+1}=-\sum_{j=1}^{n}k_{j},\;$ and $\hat{f}\left(t\right)=\frac{1}{{\left(2\pi \right)}^{n/2}}\,\int_{R^{n}}f\left(x\right)e^{-i\,t\cdot x}\,dx,\;\;t=\left(t_{1},\ldots ,t_{n}\right).$

The proof of the Gaussian fluctuation for $\left(S_{N}\left(f\right)-ES_{N}\left(f\right)\right)/\sqrt{N}$ presented in [40] relies on a detailed study of the joint cumulants $\kappa_{p}^{\left(N\right)}\left(k_{1},\ldots ,k_{p}\right),\;p>1,$ for the arguments $k_{i}=O\left(N\right),\;1\leq i\leq p.\;$ It is combinatorial in nature. One of the key ingredients of the proof is the fact that joint cumulants scale with N. Namely,

(32) $$\begin{array}{c} c_{p}\left(t_{1},\ldots ,t_{p}\right):=\frac{1}{N}\,\kappa_{p}^{\left(N\right)}\left(t_{1}\,N,\ldots ,t_{p}\,N\right) \end{array}$$

does not depend on N. Moreover, it is a piece-wise linear, bounded function of $t_{1},\ldots ,t_{p}$ on the hyperplane $t_{1}+\ldots +t_{p}=0$ (it is identically zero when $t_{1}+\ldots +t_{p}\neq 0$).

**Lemma 3**  ([40])**.** *Rescaled joint cumulants $c_{p}$ defined in (32) can be written for p>1 and $\sum_{i=1}^{p}t_{i}=0$ as*

(33) $$\begin{array}{c} c_{p}\left(t_{1},\ldots ,t_{p}\right)=\sum_{m=1}^{p}\frac{{\left(-1\right)}^{m}}{m}\,\sum_{\begin{array}{c} (p_{1},\ldots ,p_{m}):\\ p_{1}+\ldots +p_{m}=p,\;p_{1},\ldots ,p_{m}\geq 1 \end{array}}\frac{1}{p_{1}!\cdots p_{m}!}\,\sum_{\sigma \in S_{p}}\,j\left(p_{1},\ldots ,p_{m};t_{\sigma (1)},\ldots ,t_{\sigma (p)}\right), \end{array}$$

*where $j(p_{1},\ldots ,p_{m};t_{1},\ldots ,t_{p})$ is defined for positive integers $p_{1},\ldots ,p_{m},\;\;p_{1}+\ldots +p_{m}=p,$ and real numbers $t_{1},\ldots ,t_{p},$ as*

(34) $$\begin{array}{cc} \; & j(p_{1},\ldots ,p_{m};t_{1},\ldots ,t_{p}):=\\ \; & \min\left(1,\;\max(0,\sum_{i=1}^{p_{1}}t_{i},\sum_{i=1}^{p_{1}+p_{2}}t_{i},\ldots ,\sum_{i=1}^{p_{1}+\ldots +p_{m-1}}t_{i})+\max(0,\sum_{i=1}^{p_{1}}\left(-t_{i}\right),\ldots ,\sum_{i=1}^{p_{1}+\ldots +p_{m-1}}\left(-t_{i}\right))\right). \end{array}$$

*Moreover, the following holds:*

*(i)*  $c_{p}\left(t_{1},\ldots ,t_{p}\right),\;p>1,\;\;is\;a\;bounded\;symmetric\;\mathrm{piece-wise}\;linear\;function\;on\;\;\sum_{i=1}^{p}t_{i}=0.$
*(ii)*  $c_{p}\left(t_{1},\ldots ,t_{p}\right)=0\;if\;\;p>1\;\;and\;\;\sum_{i=1}^{p}t_{i}\neq 0.$
*(iii)*  $c_{1}\left(0\right)=1\;\;and\;\;c_{1}\left(t\right)=0\;\;for\;\;t\neq 0.$

We finish this section with a discussion of the multivariate linear statistics for non-smooth functions. For simplicity, we assume that n=1 and restrict our attention to the global regime ($L_{N}=1).$ Then,

(35) $$S_{N}\left(f\right)=\sum_{i,j=1}^{N}f\left(\theta_{i}-\theta_{j}\right).$$

In [45], we proved that if *f* is an even real-valued function on the unit circle such that both $f^{\prime }$ belongs to $L^{2}\left(T\right)$, then

(36) $$S_{N}\left(f\right)-ES_{N}\left(f\right)\overset{\;D\;}{\to }\,2\,\sum_{m=1}^{\infty }{\hat{f}}_{m}\,m\,\left(\varphi_{m}-1\right),$$

where $\varphi_{m}$ are i.i.d. exponential random variables with $E(\varphi_{m})=1$. We note that the condition $\sum_{k}{\left|\hat{f_{k}}\right|}^{2}\,k^{2}<\infty$ is necessary and sufficient for (36) to hold. If the series $\sum_{k}{\left|\hat{f_{k}}\right|}^{2}\,k^{2}$ is slowly diverging so that the sequence of partial sums $V_{N}=\sum_{|k|\leq N}{\left|\hat{f_{k}}\right|}^{2}\,k^{2}$ is slowly varying in the sense of Karamata ([57]), then the Central Limit Theorem holds ([46]):

$$\frac{S_{N}\left(F\right)-ES_{N}\left(f\right)}{\sqrt{V_{N}}}\overset{\;D\;}{\to }N\left(0,2\right).$$

The case of the linearly growing variance will be studied in the next section, where we will consider a class of even test functions *f* for which ${\hat{f}}_{k}\,k\to C\neq 0$ as k→∞. The values of the cumulant function $\kappa_{p}^{\left(N\right)}\left(k_{1},\ldots ,k_{p}\right)$ for $k_{i}=O\left(N\right),\;1\leq i\leq p$ play an important role in the analysis.

## 3. Results

We study the Circular Unitary Ensemble of random matrices (11). In particular, we are interested in the joint distribution of the traces of high powers of a unitary matrix *V*. As before, we denote by $\kappa_{p}^{\left(N\right)}\left(k_{1},\ldots ,k_{p}\right)$ the joint cumulants of $\mathrm{Tr}V^{k_{1}},\ldots ,\mathrm{Tr}V^{k_{p}},\;1\leq i\leq p.\;$ In what follows, $k_{i}=O\left(N\right),\;1\leq i\leq p,\;$ and we are looking for scenarios when the joint cumulants vanish.

We start by considering the joint cumulants of $\mathrm{Tr}V^{k}\;$ and $\;\mathrm{Tr}V^{-k}$ for k≥N. While the distribution in this case is well understood (see, e.g., [58]), it is instructive to consider the cumulant approach that will be further expanded later in this section. We are interested in the values of the joint cumulants $\kappa_{p}^{\left(N\right)}\left(k_{1},\ldots ,k_{p}\right),\;p>1,$ where

$$k_{1}=\ldots =k_{q}=k\;and\;k_{q+1}=\ldots =k_{p}=-k,\;0\leq q\leq p,\;k\geq N.$$

It follows from Lemma 1 that $\kappa_{p}^{\left(N\right)}\left(k,\ldots ,k,-k,\ldots ,-k\right)=0$ unless *p* is even and p=2⁢q. We then have $k_{1}=\ldots k_{q}=k$ and $k_{q+1}=\ldots =k_{2\,q}=-k.\;$ We will use the Formula (26). Since k≥N, one has

(37) $$\min\left(N,\;\max(0,\sum_{i\in B_{1}}k_{i},\ldots ,\sum_{i\in B_{1}\cup \ldots \cup B_{m-1}}k_{i})+\max(0,-\sum_{i\in B_{1}}k_{i},\ldots ,-\sum_{i\in B_{1}\cup \ldots \cup B_{m-1}}k_{i})\right)=N$$

unless each subset $B_{j}$ has an equal number of elements equal to *k* and −k, in which case the l.h.s. of (37) is zero. A routine combinatorial analysis produces

(38) $$\kappa_{2\,q}^{\left(N\right)}\left(k,\ldots ,k,-k,\ldots ,-k\right)=N\,\sum_{m=1}^{q}\frac{{\left(-1\right)}^{m-1}}{m}\,\sum_{\begin{array}{c} (q_{1},\ldots ,q_{m}):\\ q_{1}+\ldots +q_{m}=q,\;q_{1},\ldots ,q_{m}\geq 1 \end{array}}{\left(\frac{q!}{q_{1}!\cdots q_{m}!}\right)}^{2}.$$

Therefore, the cumulant generating function $C_{N}\left(x,y\right)$ can be written as

(39) $$\log\left(E\exp(x\,\mathrm{Tr}V^{k}+y\,\mathrm{Tr}V^{-k})\right)=N\,\log\left(\sum_{m=0}^{\infty }\frac{1}{{\left(m!\right)}^{2}}{\left(x\,y\right)}^{m}\right)=N\,\log\left(I_{0}\left(\sqrt{4\,x\,y}\right)\right),$$

where

(40) $$I_{0}\left(u\right)=\frac{1}{2\,\pi }\,\int_{0}^{2\,\pi }e^{u\,\cos\theta }\,d\theta =\sum_{m=0}^{\infty }\frac{1}{{\left(m!\right)}^{2}\,2^{2m}}\,u^{2\,m}$$

is the modified Bessel function of the first kind.

A more elementary way to derive (39) relies on the observation by Rains ([58]) that the *k*th powers of the eigenvalues of *V* are i.i.d. random variables uniformly distributed on the unit circle provided k≥N. Thus, $\mathrm{Tr}V^{k},$ for k≥N, is given by the sum of *N* i.i.d. uniform random variables on the unit circle. The traces of different powers are still correlated for finite N. However, a significant portion of the joint cumulants vanishes.

**Proposition 1.**  *Let $s_{i},\;1\leq i\leq l$ be l>1 distinct positive integers and $a_{i},b_{i}$ be non-negative integers such that $a_{i}+b_{i}\geq 1$ for all i. Suppose that*

(41) $$|\sum_{i=1}^{l}n_{i}\,s_{i}|\geq N\;\;\forall \;n_{1},\ldots ,n_{l}\in Z:-b_{i}\leq n_{i}\leq a_{i},\;\;1\leq i\leq l,\;\;\sum_{i=1}^{l}\left|n_{i}\right|>0.$$

*Let $p=\sum_{1}^{l}\left(a_{i}+b_{i}\right)$ and $\left(k_{1},\ldots ,k_{p}\right)\in Z^{p}$ be such that the first $a_{1}$ coordinates of the vector are equal to $s_{1},\;$ the next $b_{1}$ coordinates are equal to $-s_{1},\;$ the further next $a_{2}$ coordinates are equal to $s_{2},$ the following $b_{2}$ coordinates are equal to $-s_{2},\;$ etc, …, and the last $b_{l}$ coordinates are equal to $-s_{l}.$*

*Then,*

(42) $$\kappa_{p}^{\left(N\right)}\left(k_{1},\ldots ,k_{p}\right)=0.$$

*Moreover, for $\;0\leq n_{i}\leq a_{i},\;\;0\leq m_{i}\leq b_{i},\;1\leq i\leq l\;$, one has*

(43) $$E\left[\prod_{i=1}^{l}{\left(\mathrm{Tr}V^{s_{i}}\right)}^{n_{i}}\,{\left(\mathrm{Tr}V^{-s_{i}}\right)}^{m_{i}}\right]=\delta_{nm}\,\prod_{i=1}^{l}E\left[|\sum_{j=1}^{N}\varphi_{j}{|}^{2\,n_{i}}\right],$$

*where $\left\{\varphi_{i}\right\}$ are i.i.d. uniform random variables on the unit circle, and $\delta_{nm}$ is the delta symbol.*

**Proof of Proposition 1.**  While the proof does not depend on the value of l, we will assume that l=2 in order to simplify the notations. It follows from (41) that the joint cumulant is zero unless

(44) $$a_{i}=b_{i},\;\;1\leq i\leq 2,$$

since otherwise $\sum_{1}^{p}k_{i}\neq 0.$ Therefore, from now on, we can assume that (44) holds. As before, we will use (26) to compute the joint cumulants. We call a subset $B_{j}$ balanced if the number of times $s_{1}$ appears in it is equal to the number of times $-s_{1}$ appears in it, and the same holds for $s_{2}$ and $-s_{2}.$ It follows from (41) that unless unless each subset $B_{j}$ is balanced, the l.h.s. of (37) is N. Otherwise, the l.h.s. of (37) is zero. We obtain that for $a_{1}=b_{1}=c$ and $a_{2}=b_{2}=d$,

$$\begin{array}{c} \kappa_{p}^{\left(N\right)}\left(s_{1},\ldots ,s_{1},-s_{1},\ldots ,-s_{1},s_{2},\ldots ,s_{2},-s_{2},\ldots ,-s_{2}\right)=N\,\sum_{m\geq 1}\frac{{\left(-1\right)}^{m-1}}{m}\\ \,\sum_{\begin{array}{c} c_{1}+\ldots +c_{m}=c,\;c_{1},\ldots c_{m}\geq 0\\ d_{1}+\ldots +d_{m}=d,\;d_{1},\ldots d_{m}\geq 0\\ c_{i}+d_{i}\geq 1,\;1\leq i\leq m \end{array}}{\left(\frac{c!}{c_{1}!\cdots c_{m}!}\right)}^{2}\,{\left(\frac{d!}{d_{1}!\cdots d_{m}!}\right)}^{2},\;\;\;\;\;\;\;\;p=2\,\left(c+d\right). \end{array}$$

Therefore, the coefficient in front of ${\left(xy\right)}^{c}\,{\left(zw\right)}^{d}$ in the cumulant generating function

$$C_{N}\left(x,y,z,w\right):=\log\left(E\exp(x\,\mathrm{Tr}V^{s_{1}}+y\,\mathrm{Tr}V^{-s_{1}}+z\,\mathrm{Tr}V^{s_{2}}+w\,\mathrm{Tr}V^{-s_{2}})\right)$$

coincides with the corresponding coefficient in the power series expansion of

$$\begin{array}{ccc} \; & \; & N\,\log\left(\sum_{m=0}^{\infty }\frac{1}{{\left(m!\right)}^{2}}{\left(x\,y\right)}^{m}\,\sum_{m=0}^{\infty }\frac{1}{{\left(n!\right)}^{2}}{\left(z\,w\right)}^{n}\right)=N\,\log\left(I_{0}\left(\sqrt{4\,x\,y}\right)\,I_{0}\left(\sqrt{4\,z\,w}\right)\right)\\ \; & \; & =N\,\log\left(I_{0}\left(\sqrt{4\,x\,y}\right)\right)+N\,\log\left(I_{0}\left(\sqrt{4\,z\,w}\right)\right)\\ \; & \; & =\log\left(E\exp(x\,\sum_{j=1}^{N}\phi_{j}+y\,\sum_{j=1}^{N}\bar{\phi_{j}}+z\,\sum_{j=1}^{N}\eta_{j}+w\,\sum_{j=1}^{N}\bar{\eta_{j}})\right), \end{array}$$

where $\left\{\phi_{i}\right\},\;\left\{\eta_{i}\right\}$ are i.i.d. uniform random variables on the unit circle, and $\bar{\phi }$ denotes the complex conjugate. The identity (43) follows from the derived identity for the cumulants and the Formula (18), expressing moments in terms of cumulants. □

To illustrate the utility of the cumulant approach, we formulate and prove below the Central Limit Theorem for pair-counting statistics for a class of non-smooth test functions. We consider

(45) $$S_{N}\left(f\right)=\sum_{i,j=1}^{N}f\left(\theta_{i}-\theta_{j}\right),$$

where $\left(\theta_{1},\ldots ,\theta_{N}\right)$ is a random CUE point configuration, and *f* is an even real-valued function on the unit circle such that

(46) $${\hat{f}}_{k}\,k\to C\neq 0\;\;\mathrm{as}\;k\to \infty .$$

**Remark 6.**  *f(θ)=log|sin(θ/2)| satisfies the above test function requirements.*

**Theorem 4.**  *Let f be a real even function on the unit circle, satisfying (46). Then,*

(47) $$\frac{S_{N}\left(f\right)-ES_{N}\left(f\right)}{\sqrt{N}}\overset{\;D\;}{\to }N\left(0,\sigma^{2}\left(C\right)\right),$$

*where the limiting variance can be computed as*

(48) $$\begin{array}{c} \sigma^{2}\left(C\right)=4\,C^{2}\,\left(3-2\,\int_{0}^{1}\frac{1}{y}\,\left((1+1/y)\,\log(1+y)-1\right)\,dy\right) \end{array}$$

(49) $$\begin{array}{c} -4\,C^{2}\,\left(2\,\int_{0}^{1}\;\frac{1}{y}\,\left(1+y\right)\,\log\left(1+y\right)\,dy+\int_{0}^{1}\frac{1-y}{y}\,\log\left(1-y\right)\,dy\right). \end{array}$$

**Proof of Theorem 4.**  We start with the following formula for the variance of $S_{N}\left(f\right)$:

**Proposition 2**  ([45])**.** *Let f be a real even function on the unit circle such that $f\in L^{2}\left(T\right).$ Then,*

(50) $$\begin{array}{c} Var\left[S_{N}\left(f\right)\right]=4\left(\sum_{1\leq s\leq N-1}s^{2}{\left(\hat{f}\left(s\right)\right)}^{2}+N^{2}\sum_{N\leq s}{\left(\hat{f}\left(s\right)\right)}^{2}-N\sum_{\begin{array}{c} N\leq s \end{array}}{\left(\hat{f}\left(s\right)\right)}^{2}\right) \end{array}$$

(51) $$\begin{array}{c} -4\left(\sum_{\begin{array}{c} 1\leq s,t\\ 1\leq |s-t|\leq N-1\\ N\leq \max(s,t) \end{array}}\left(N-|s-t|\right)\hat{f}\left(s\right)\hat{f}\left(t\right)\;+\sum_{\begin{array}{c} 1\leq s,t\leq N-1\\ N+1\leq s+t \end{array}}\left(\left(s+t\right)-N\right)\hat{f}\left(s\right)\hat{f}\left(t\right)\right). \end{array}$$

Since the test function *f* satisfies (46), we have

$$\sum_{1\leq s\leq N-1}s^{2}{\left(\hat{f}\left(s\right)\right)}^{2}=N\,\left(1+o\left(1\right)\right),\;\;\sum_{N\leq s}{\left(\hat{f}\left(s\right)\right)}^{2}=\frac{1}{N}+O\left(\frac{1}{N^{2}}\right),$$

and the two double sums in (51), up to a negligible error, are equal to the Riemann sums of converging integrals. As a result,

(52) $$\mathrm{Var}\left[S_{N}\left(f\right)\right]=4\,C^{2}\,N\,\left(2-\int_{D_{1}}\left(1-|x-y|\right)\,\frac{1}{x\,y}\,dx\,dy-\int_{D_{2}}\left(x+y-1\right)\,\frac{1}{x\,y}\,dx\,dy\right)\left(1+o\left(1\right)\right),$$

where

$$D_{1}=\left\{\left(x,y\right):x,y>0,|x-y|\leq 1,\;\max\left(x,y\right)\geq 1\right\},D_{2}=\left\{\left(x,y\right):0<x,y\leq 1,x+y\geq 1\right\}.$$

The integrals in (52) can be trivially simplified as

(53) $$\begin{array}{c} \int_{D_{1}}\left(1-|x-y|\right)\,\frac{1}{x\,y}\,dx\,dy=-2+2\,\int_{0}^{1}\;\frac{1}{y}\,\left(1+y\right)\,\log\left(1+y\right)\,dy\\ +2\,\int_{1}^{\infty }\frac{1}{y}\,\left((1+y)\,\log(1+1/y)-1\right)\,dy,\\ \int_{D_{2}}\left(x+y-1\right)\,\frac{1}{x\,y}\,dx\,dy=1+\int_{0}^{1}\frac{1-y}{y}\,\log\left(1-y\right)\,dy, \end{array}$$

which leads to (48) and (49).

To prove the theorem, we will study asymptotics of the higher moments of $S_{N}\left(f\right).$ We start by truncating f. Namely, we replace it by $\tilde{f}\left(x\right)=\sum_{|k|\leq N\,\log N}\hat{f_{k}}\,e^{i\,k\,x}.$ It follows from (50) and (51) that $\mathrm{Var}\left[S_{N}\left(f\right)-S_{N}\left(\tilde{f}\right)\right]=\mathrm{Var}\left[S_{N}\left(f-\tilde{f}\right)\right]=o\left(N\right).$ Therefore, without a loss of generality, we can assume that the Fourier coefficients ${\hat{f}}_{k}$ are zero for |k|>N⁢logN. Whenever it does not lead to ambiguity, we will use the notation *f* for the truncated version of a test function. Recall that

(54) $$S_{N}\left(f\right)=\sum_{k}\hat{f_{k}}{\left|T_{N,k}\right|}^{2}=\sum_{k}\hat{f_{k}}T_{N,k}\,T_{N,-k},$$

where we use the notation $T_{N,k}=\mathrm{Tr}V^{k}.\;$ Let us fix a positive integer m>2. We have

$$E{\left(S_{N}\left(f\right)-ES_{N}\left(f\right)\right)}^{m}=\sum_{k_{1},\ldots ,k_{m}\in Z}\,{\hat{f}}_{k_{1}}\cdots {\hat{f}}_{k_{m}}\;E\left[\prod_{i=1}^{m}\left(T_{N,k_{i}}\,T_{N,-k_{i}}-ET_{N,k_{i}}\,T_{N,-k_{i}}\right)\right].$$

Let us denote $k_{m+i}=-k_{i},\;1\leq i\leq m.\;$ Applying

**Lemma 4.**  *9.2 from [45] (see also Lemma 4.1 in [40]), one rewrites*

(55) $$E\left[\prod_{i=1}^{m}\left(T_{N,k_{i}}\,T_{N,-k_{i}}-ET_{N,k_{i}}\,T_{N,-k_{i}}\right)\right]=E\left[\prod_{i=1}^{m}\left(T_{N,k_{i}}\,T_{N,k_{m+i}}-ET_{N,k_{i}}\,T_{N,k_{m+i}}\right)\right]$$

as $\sum_{\pi }^{*}\prod_{B\in \pi }\kappa \left(T_{N,k_{i}}:i\in B\right),$ where the sum is over all partitions π of {1,…,2m} that do not contain atoms and two-element subsets of the form {i,i+m},i=1,…,m. Therefore,

(56) $$E{\left(S_{N}\left(f\right)-ES_{N}\left(f\right)\right)}^{m}=\sum_{\pi }^{*}\;\sum_{k_{1},\ldots ,k_{m}\neq 0}\,{\hat{f}}_{k_{1}}\cdots {\hat{f}}_{k_{m}}\prod_{B\in \pi }\kappa \left(T_{N,k_{i}}:i\in B\right).$$

*For a given partition π, denote*

(57) $$\Sigma_{\pi }=\sum_{k_{1},\ldots ,k_{m}\neq 0}\,{\hat{f}}_{k_{1}}\cdots {\hat{f}}_{k_{m}}\prod_{B\in \pi }\kappa \left(T_{N,k_{i}}:i\in B\right)$$

*and*

(58) $$S_{\pi }=\sum_{\begin{array}{c} k_{1},\ldots ,k_{m}\neq 0\\ |k_{i}|\leq N\,\log N \end{array}}\,\frac{1}{|k_{1}|}\cdots \frac{1}{|k_{m}|}\prod_{B\in \pi }\,\left[\min(N,\sum_{i\in B}|k_{i}\left|\right)\,1_{\sum_{i\in B}k_{i}=0}\right].$$

*Clearly,*

(59) $$\Sigma_{\pi }=O\left(S_{\pi }\right).$$

*Following [40,45], we denote*

(60) [i]:={i,i+m},1≤i≤m,

*and introduce the equivalence relation $\;{∼}_{\pi }\;$ on the set {[1],…,[m]}:*

(61) $$\left[i\right]{∼}_{\pi }\left[j\right]$$

*if and only if there is a sequence of blocks $B_{1},\ldots ,B_{q},\;q\geq 1,$ of the partition π such that*

(62) $$B_{1}\cap \left[i\right]\neq \emptyset ,\;B_{1}\cap \left[i_{1}\right]\neq \emptyset ;\;B_{2}\cap \left[i_{1}\right]\neq \emptyset ,\;B_{2}\cap \left[i_{2}\right]\neq \emptyset ;\ldots ;B_{q}\cap \left[i_{q-1}\right]\neq \emptyset ,\;B_{q}\cap \left[j\right]\neq \emptyset ,$$

*for some $1\leq i_{1},\ldots ,i_{q-1}\leq m.\;$ We call a partition π optimal if the cardinality of every equivalence class is 2. We claim that for each optimal partition π, the corresponding sum $\Sigma_{\pi }=\sigma^{m}\,N^{m/2}\,\left(1+o\left(1\right)\right),\;$ and for each suboptimal partition, $\Sigma_{\pi }=o\left(N^{m/2}\right).$*

*The first part of the claim immediately follows from the variance computations above, as the m−dimensional sum (57) then factorizes into the product of m/2 two-dimensional sums, each equal $\sigma^{2}\,N\,\left(1+o\left(1\right)\right).\;$*

*Next, we will show that the suboptimal partitions give negligible contributions $o(N^{m/2}).\;$ One of the key ingredients is the following upper bound on $S_{\pi }$*.

**Lemma 5.**  *Let π be a partition of {1,…,2m} that does not contain atoms and two-element subsets of the form {i,i+m},i=1,…,m. Then,*

(63) $$S_{\pi }=\Omega \left(N^{m/2}\right),$$

*i.e., $S_{\pi }\leq N^{m/2}\,{\left(\log N\right)}^{d}$ for some d and all sufficiently large N. Moreover, if π contains at least one block of size less than 4, then*

(64) $$S_{\pi }=\Omega \left(N^{\frac{m-1}{2}}\right).$$

**Proof of Lemma 4.**  The proof uses mathematical induction on m. Without a loss of generality, we can assume that the equivalence relation ${∼}_{\pi }$ has only one equivalence class. Otherwise, the sum $S_{\pi }$ factorizes over the equivalence classes, and the argument is applicable to each equivalence class separately.

If *π* does not contain an equivalence class of size less than 4, the bound (63) follows since the number of terms in the product on the r.h.s. of (58) is not bigger than m/2, each cumulant is O(N), and the partial sums of the harmonic series grow logarithmically.

Suppose now that *π* contains a block of size 2, say $B_{1}=\left\{i,j+m\right\},1\leq i\neq j\leq m.$ Then, {i+m,j} cannot be a block of the partition. Consider the block of the partition that contains i+m. Without a loss of generality, we can assume that this block is $B_{2},$ i.e., $i+m\in B_{2}.\;$ Construct a partition $\pi^{\prime }$ of the set {1,…,2m}\{i,i+m}, where we discard $B_{1}$ and replace i+m in $B_{2}$ by j+m. It follows from the construction that $S_{\pi }\leq S_{\pi^{\prime }}$ and by the inductive hypothesis, $\;S_{\pi^{\prime }}=\Omega \left(N^{\frac{m-1}{2}}\right).$

Now, suppose that *π* has a block of size 3, say $B_{1}=\left\{i,j,l+m\right\},\;1\leq i,j,l\leq m,\;l\neq i,j.\;$ Consider the block of π that contains l, say $l\in B_{2}.\;$ Construct a partition $\pi^{\prime }$ of the set {1,…,2m}\{l,l+m}, where we discard $B_{1}$ and replace *l* in $B_{2}$ by *i* and j. Then, $S_{\pi }\leq S_{\pi^{\prime }}$ and, again, applying the inductive hypothesis, $S_{\pi^{\prime }}=\Omega \left(N^{\frac{m-1}{2}}\right).$ □

**Remark 7.**  *A similar argument shows that if π contains a block of size *4* of the form*

*{i,j,l,l+m},1≤i,j,l≤m, then $S_{\pi }=\Omega \left(N^{\frac{m-1}{2}}\right).$*

*Finally, if π contains a block of size *4* of the form $B_{1}=\left\{i,j,n+m,l+m\right\},\;1\leq i,j,n,l\leq m,\;$ such that {i,j}≠{n,l}, we consider two cases, namely, $|k_{n}\left|>\right|k_{l}|$ and $|k_{n}\left|\leq \right|k_{l}|.$ In the first case, we construct a partition $\pi^{\prime }$ of the set {1,…,2m}\{n,n+m}, where we discard $B_{1}$ and replace n in its block by i,j, and l+m. In the second case, we do the same procedure with l instead of n. In both cases, the sum is bounded from above by a sum of dimension one less, and we again obtain $S_{\pi }=\Omega \left(N^{\frac{m-1}{2}}\right)$ by the inductive assumption.*

*Now we are ready to finish the proof of the theorem. Recall that we could assume without a loss of generality that the equivalence relation ${∼}_{\pi }$ has only one equivalence class. Note that (59) and (63), Lemma 4 and Remark 7 prove $\Sigma_{\pi }=o\left(N^{m/2}\right)$ for all suboptimal partitions π, except the ones that comprise the blocks of size *4* of the form {i,j,j+m,l+m},1≤i,j,l≤m,i≠l. Since $k_{i}+k_{j}+k_{j+m}+k_{l+m}=0,$ and $k_{j+m}=-k_{j},$ we obtain $k_{l+m}=-k_{i}.\;$ It follows from the direct computations (see, e.g., [40]) that*

(65) $$\kappa_{4}^{\left(N\right)}\left(k_{i},k_{j},-k_{j},-k_{i}\right)=\left\{\begin{array}{cc} 0, & \;if\;1\leq |k_{i}\left|=\right|k_{j}|\leq N/2,\\ N-2|k_{i}|, & \;if\;N/2<|k_{i}\left|=\right|k_{j}|\leq N,\\ -N, & if\;|k_{i}\left|=\right|k_{j}|\geq N,\\ \left|\right|k_{i}\left|-\right|k_{j}\left|\right|-N, & \;if\;1\leq \left|\right|k_{i}\left|-\right|k_{j}\left|\right|\leq N-1,\;N\leq \max(|k_{i}\left|,\right|k_{j}\left|\right),\\ N-|k_{i}\left|-\right|k_{j}|, & if\;1\leq |k_{i}\left|\neq \right|k_{j}\left|\leq N-1,N+1\leq \right|k_{i}\left|+\right|k_{j}|,\\ 0, & else. \end{array}\right..$$

*In particular, $|\kappa_{4}^{\left(N\right)}\left(k_{i},k_{j},-k_{j},-k_{i}\right)\left|\leq \min(\right|k_{i}\left|,\right|k_{j}\left|\right).$ Without a loss of generality, we can assume that $\pi =\{B_{1},\ldots ,B_{m/2}\},$ where*

(66) $$\begin{array}{c} B_{1}=\left\{1,2,2+m,3+m\right\},\;B_{2}=\left\{3,4,4+m,5+m\right\},\ldots , \end{array}$$

(67) $$\begin{array}{c} B_{m/2-1}=\left\{m-3,m-2,2\,m-2,2\,m-1\right\},\;B_{m/2}=\left\{m-1,m,2\,m,m+1\right\}. \end{array}$$

*Then,*

(68) $$\Sigma_{\pi }=\sum_{k_{1},\ldots ,k_{m}\neq 0}\,{\hat{f}}_{k_{1}}\cdots {\hat{f}}_{k_{m}}\prod_{i=1}^{m/2}\kappa_{4}^{\left(N\right)}\left(k_{2\,i-1},k_{2\,i},-k_{2\,i},-k_{2\,i+1}\right).$$

*Since the fourth cumulant function vanishes unless the sum of the arguments is zero, one has $k_{1}=k_{3}=k_{5}=\ldots =k_{m-1}.\;$ Moreover, the fourth cumulant function vanishes unless the sum of the absolute values of the arguments is at least 2⁢N. Therefore, $|k_{1}\left|+\right|k_{2\,i}|\geq N,$1≤i≤m, and*

(69) $$|\Sigma_{\pi }|\leq Const\,\sum_{\begin{array}{c} k_{1}\neq 0,\\ |k_{1}|\leq N\,\log N \end{array}}\,\sum_{\begin{array}{c} k_{2},k_{4},k_{6},\ldots ,k_{m}\neq 0\\ |k_{2\,i}|\leq N\,\log N \end{array}}\,\frac{1}{|k_{1}{|}^{m/2}}\,\prod_{i=1}^{m/2}\frac{1}{|k_{2\,i}|}\prod_{i=1}^{m/2}\min(|k_{1}\left|,\right|k_{2\,i}\left|\right)\,1_{|k_{1}\left|+\right|k_{2\,i}|\geq N}.$$

*Splitting the summation with respect to $k_{1}$ into two parts, corresponding to $|k_{1}|\leq \sqrt{N}$ and $|k_{1}|>\sqrt{N}$ correspondingly, one arrives at the bound:*

$$|\Sigma_{\pi }|\leq 2\times Const\,\left(\sum_{0<k_{1}\leq \sqrt{N}}1\,\sum_{\begin{array}{c} k_{2},k_{4},k_{6},\ldots ,k_{m}\neq 0\\ |k_{2\,i}|\leq N\,\log N \end{array}}\,\prod_{i=1}^{m/2}\frac{1}{|k_{2\,i}|}+\sum_{|k_{1}|\geq \sqrt{N}}\frac{1}{|k_{1}{|}^{m/2}}\,\sum_{\begin{array}{c} k_{2},k_{4},k_{6},\ldots ,k_{m}\neq 0\\ |k_{2\,i}|\leq N\,\log N \end{array}}1\right).$$

*The right-hand side is $=o(N^{m/2})$ for m>2. Therefore, we have shown that $\Sigma_{\pi }=o\left(N^{m/2}\right)$ for all suboptimal partitions.*

*If m=2⁢q+1 is odd, then cleraly there are no optimal partitions, which implies*

(70) $$E{\left(S_{N}\left(f\right)-ES_{N}\left(f\right)\right)}^{2\,q+1}=o\left(N^{q+1/2}\right).$$

*If m=2⁢q is even, then there are exactly (2⁢q−1)!! optimal partitions, and for each such partition π, one has $\Sigma_{\pi }=\sigma^{2\,q}\left(C\right)\,N^{q}\,(1+o\left(1\right).$ When we combine these results together, we obtain*

(71) $$E{\left(S_{N}\left(f\right)-ES_{N}\left(f\right)\right)}^{2\,q}=\left(2\,q-1\right)!!\,\sigma^{2\,q}\left(C\right)\,N^{q}\,(1+o\left(1\right).$$

*The bounds (70) and (71) finish the proof.* □

## 4. Discussion

We have discussed several applications of the cumulant technique in Random Matrix Theory, specifically for the ensembles with determinantal *k*-point correlation functions. The suggested approach to the fluctuations of multivariate linear statistics of the eigenvalues of random unitary matrices can be extended to other classes of test functions and other classical groups.

## Data Availability

Data sharing not applicable to this article, as no datasets were generated or analyzed during the current study.
